# Remarks on Cancer in Connection with the Vital Nervous System and the Blood

**Published:** 1867-03

**Authors:** John O’Reilly

**Affiliations:** 114 West Fourth Street, (Washington Square South); New York


### REMARKS ON CANCER IN CONNECTION WITH THE VITAL NERVOUS SYSTEM AND THE BLOOD.

### OBSERVATIONS OF DR. PAUL BROCA, SURGEON L'HOSPITAL SAINT ANTOINE, PARIS.

By JOHN O'REILLY.

"When the yellow, straw-colored appearance" of the skin manifests itself, the operation for cancer should not be performed; because the blood is then infected by the cancerous poison (*suc cancereux*), and the vital nervous system is brought under its influence; the vital glands in which the arteries terminate and veins commence, are now dyed with the coloring of the cancer contained in the blood, precisely as they are dyed by the bile in the case of jaundice, or as they become dyed a bluish color by the oxide of silver in the patient who has been treated for epilepsy with nitrate of silver.* Let me quote from Dr. Broca's work on Tumors, page 332.

"In other words, we admit that the elements of the disease succeed each other in the following order:

*This is the best proof that can be advanced that the arteries terminate in glands, and that the veins commence in capillaries from the glands. The arterial blood, containing the silver, is carried as far as the vital glands, where the silver is arrested in its progress through the glands, and where, being exposed to the light, forms an oxide of silver, that gives the bluish tinge to the skin; the silver thus being literally sifted out of the blood. The necessity for understanding this matter well, will be gleaned from what Dr. Broca says, at page 370:

"Nos connaissances ne sont ni plus ni moins avancées sur ce point qu'elles ne le sont a l'égard de la cause immédiate des boutons varoliques, des ulcérations de la morve, des éruptions folliculeuses de la fièvre typhoïde et du choléra, et des manifestations multipées de la syphilis constitutionelle, lesions anatomiques développées, comme les tumeurs par infection sous l'influence d'un état général de l'économie."

"1. The *diathese* or hereditary predisposition produces the primitive cancer.

"2. That produces the infection.

"3. The infection produces the multiplied secondary tumors, the cachexia and death."

If an operation for cancer is decided on, what is the most feasible time for performing it, or when is the operation likely to be followed by temporary success?

Let it be here remembered that Dr. Broca has conclusively established (page 335) that cancer is an hereditary (*diathese*) disease, and that the operation can never be performed with the same certainty of ultimate success as an operation for lithotomy or hernia, when the patient is operated on before the poison is absorbed into the blood from the part or organ affected, and before the vital nervous system is contaminated. "The experiments performed by M. Langenbeck (page 348) illustrate this point, that the *suc cancereux*, when injected into a vein, is capable of producing the disease in a dog"—so in like manner the *suc cancereux*, when taken into the venous circulation of a man, is capable of producing cancer in any part or organ of the body.

"When an individual," says Dr. Broca (page 333), "born of sound parents, becomes the subject of cancer—for example at the age of forty years—and dies in about three years in the natural progress of the disease, after having presented, during the last year, all the signs of cancerous infection," etc., etc.

It will be seen from these remarks of Dr. Broca, that the poison is not absorbed, or rather that the blood, and consequently the vital nervous system is not poisoned until the year previous to the death of the patient, showing that the operation should be performed during the first year. In corroboration of this Dr. Broca remarks (page 355): "It is well known that the circumscribed encephaloid, or what is commonly called encysted encephaloid, can exist for a long time, and acquire a very large size without infecting the constitution."

When the blood is poisoned, no person can tell, after the operation is performed and the disease locally removed, where it may next appear; as for example it may appear in the wound, or in some distant part or organ of the body;* this doctrine is

*"Relapses by repullulation," as they are termed by Dr. Broca (page 375), are the result of general poisoning of the vital nervous system, through the agency of the blood, from the part primarily affected, or through hereditary (*diathese*) taint.

In the case of a dissecting wound, the poison is first localized in the wound, as in the case of localized cancer; but after the lapse of some days, the poison strengthened by its analogy to other cases, as where a person gets a dissecting wound; after a certain time he manifests all the symptoms of being poisoned. The condition of the original wound at this period, according to the description by Professor Colles, of the Royal College of Surgeons in Ireland, of the case of Dr. Dease, given in the Dublin Hospital Reports, a vesicle existed corresponding to the wound, showing that the poison had acted on the vital nerves of the wound before being carried into the general circulation of the blood, and before poisoning the vital nerves in the heart and in the internal coat of the arteries.

When a man receives the bite of a rabid animal, the original wound may be healed, with the vital nerves at the same time poisoned; but when the *rabies* sets in, the wound will be observed to become inflamed, as has been noticed by Professor Colles in his lectures showing that the poison had been there for a certain period, remaining dormant, until it passes into the arterial circulation and poisons the vital nerves of the heart and arteries, causing death.

It is to be remarked that certain poisons act more promptly on the vital nervous system than others; as, for example, the venom of certain reptiles will kill in periods from one to three hours.

In the case of a dissecting wound, some time may elapse before the whole vital nervous system is brought under the influence of the poison; in other words, the poison may remain located in the wound twenty-four hours before it is absorbed into the blood, and consequently before it poisons the vital nervous system.

In the case of a bite of a rabid animal, weeks or months may pass by before the poison is carried from its original location in the wound to the vital nervous system through the blood. In is propagated to distant parts of the body; as, for instance, in the case of Mr. Dease, who received a dissecting wound in the thumb, on the 13th of February, 1819, at 1 o'clock P.M. A vesicle of the size of a small pea, was observed at 9 P.M., on Tuesday, 16th; on Sunday, 21st, a swelling was observed on the anterior part of the right arm; and Mr. D. died at 10 P.M. the same evening. On Monday morning, Mr. Colles examined the body; a vesicle of the size of a half dime, had formed on his back; the swelling had extended down the thigh; the left arm was swelled, and rather hardened from the elbow nearly up to the shoulder; the swelling was chiefly along its anterior surface, but it could also be felt all around, yet there was no redness or vesication of the limb.

Any person can see the analogy between the relapses by repullulation in the case of cancer, and the condition of remote parts in the case of the dissecting wound—the vital nervous system being poisoned in both cases, but by different poisons.

the case of a cancer, years may elapse before the poison manifests itself, by entering the blood from the parts locally affected, and brought in contact with the whole vital nervous system.

The practical deduction from all these facts is, that not a moment should be lost in removing, by the knife, the part infected, in the case of the dissecting wound, the bite of the rabid animal, the reptile, and the virus of the cancer.

What proofs are there that the poison is confined to a particular part for a certain time? The proofs afforded by the phenomena caused by the process of vaccination, and the proofs afforded by the inoculation of syphilitic poison. The local appearances in these cases are too well known to require elucidation.

Where is the poison in these cases located? In the vital glands commonly called the *rete mucosum.* What is the proof that the vaccine matter remains in the vital glands or *rete mucosum?* Because the slight wounds made in vaccination do not pass through the corion or cutis vera; the same explanation is true of the application of the syphilitic poison, as exemplified in the case of chancre, when abrasion of the cuticle takes place, leaving the *rete mucosum* exposed to the infection of poison. I will further add, in connection with these latter remarks, the case of Mr. Hutchinson, stated in the Dublin Hospital Reports (Vol. III), who, on the 1st of December, 1818, received a *slight* scratch of the knife on the outer phalanx of the right thumb while dissecting, and resulted in poisoning the wound.

To give an idea of my theory, let us suppose that a man is bitten by a reptile on the hand; the poison is carried by the absorbents from the wound into the venous circulation next to the right side of the heart; from that to the lungs; thence, after being oxygenized, to the left side of the heart; thence to the arteries all over the trunk, head and extremities, to the *rete mucosum;* thus poisoning the vital nerves of the heart and arteries, causing death. *Let it be observed the heart or arteries receive no nerves from the cerebro-spinal system.**

* The vapor of the chloroform passes into the blood through the lungs with the oxygen of the air, and is carried with the blood to the left side of the heart where it is brought in immediate contact with the internal *surface*, or membrane of the heart, and is next carried into the aorta, where it is brought in contact with the internal *surface*, or membrane of the aorta; so, in like manner, it is carried all over the whole body by the arteries, where it is brought in contact with the internal *surface*, or membrane of the arteries. The blood being charged with the chloroform in periods varying, some short, some long, weakens the powers of life, or destroys it altogether, causing the extinction of life in the vital nervous tissue, by being brought in contact with it so extensively through its *connection* with the heart and arteries.

I will now conclude by remarking that had God preserved the great M. Bichat (who died July 20, 1802, at the early age of 30 years and a few months) to the ordinary age of man, medical science would be now in a vastly higher condition of advancement than it actually is. I am now endeavoring to fill up the hiatus left by him, although not possessed of his genius, talents, or acquirements.

To understand the *modus operandi* of the chloroform, it must be remembered that the left auricle and ventricle of the heart are lined with an exceedingly thin and transparent membrane, which is reflected on the semi-lunar valves, and becomes continuous with the lining membrane, or *surface* of the aorta. The connection between the lining membrane of the heart and the muscular fibres is exceedingly intimate, as well as the valves and the elastic coat of the aorta—so much so, that it cannot be removed, except piecemeal, without breaking up the close and delicate connection which exists between them. The membrane appears to be an expansion, or termination of vessels and nerves, derived from the elastic coat; the elastic and internal coats are one.

The question now may be asked. What is the aorta connected with, externally, besides the cellular coat? With a complex net-work of vital nerves, derived from the aoratic plexus, which completely surrounds the artery. Some of these nerves *perforate* the elastic coat, and are *lost* on the internal *surface*, or membrane. The cardiac (vital) nerves, which supply the muscular fibres of the heart, are derived from the cardiac plexus, and are distributed to the muscular fibres of the heart, and terminate in the lining membrane, or *surface* of the heart. The muscular structure can be seen without dissection.

The observations made with respect to the aorta, are applicable to all the large arteries. Every anatomist knows that they are surrounded by a net-work of vital nerves, some of which are lost in the cellular coat, whilst others *perforate* the elastic coat to reach its internal *surface*, or internal coat, as it is usually called—in this way extending along the artery to the vital gland.

I have just verified the observations made with regard to the heart and aorta, by the examination of the heart and aorta of an ox, February 7, 1867. I recommend others to satisfy themselves by adopting a similar course.*

* Let any person endeavor to peel an apple without removing a portion of the pulp.

*114 West Fourth Street,*
(Washington Square South).

NEW YORK, *February 9, 1867.*

